# Prognostic Value of Cell-Surface Vimentin-Positive CTCs in Pediatric Sarcomas

**DOI:** 10.3389/fonc.2021.760267

**Published:** 2021-12-09

**Authors:** Long Dao, Dristhi Ragoonanan, Izhar Batth, Arun Satelli, Jessica Foglesong, Jian Wang, Wafik Zaky, Jonathan B. Gill, Diane Liu, Aisha Albert, Nancy Gordon, Winston Huh, Douglas Harrison, Cynthia Herzog, Eugenie Kleinerman, Richard Gorlick, Najat Daw, Shulin Li

**Affiliations:** ^1^ Department of Pediatrics, The University of Texas MD Anderson Cancer Center, Houston, TX, United States; ^2^ Department of Biostatistics, The University of Texas MD Anderson Cancer Center, Houston, TX, United States

**Keywords:** cell surface vimentin (CSV), adult and young adolescent (AYA), receiver operating characteristic curve (ROC), area under curve (AUC), circulating tumor cell (CTC), epithelial cellular adhesion molecule (EpCAM), clinical laboratory improvement amendments (CLIA).

## Abstract

**Background:**

Despite advances in care, the 5 year overall survival for patients with relapsed and or metastatic sarcoma remains as low as < 35%. Currently, there are no biomarkers available to assess disease status in patients with sarcomas and as such, disease surveillance remains reliant on serial imaging which increases the risk of secondary malignancies and heightens patient anxiety.

**Methods:**

Here, for the first time reported in the literature, we have enumerated the cell surface vimentin (CSV+) CTCs in the blood of 92 sarcoma pediatric and adolescent and young adult (AYA) patients as a possible marker of disease.

**Results:**

We constructed a ROC with an AUC of 0.831 resulting in a sensitivity of 85.3% and a specificity of 75%. Additionally, patients who were deemed to be CSV+ CTC positive were found to have a worse overall survival compared to those who were CSV+ CTC negative. We additionally found the use of available molecular testing increased the accuracy of our diagnostic and prognostic tests.

**Conclusions:**

Our findings indicate that CSV+ CTCs have prognostic value and can possibly serve as a measure of disease burden.

## Introduction

The current standard of care for patients with sarcoma includes molecular genetic testing. The purpose of this testing is often to obtain an accurate diagnosis of soft-tissue sarcoma, so as to reveal the severity of the disease and can be used in prognosis. Recent research has focused on the presence of fusion genes in sarcoma (1), which are caused by genetic instability and are accompanied by a poor prognosis. Common molecular targets include amplified or altered *TP53* and *EWSR1* gene fusions in Ewing sarcoma (2, 3). Information from molecular genetic testing could be combined with other liquid biopsy techniques to obtain a more accurate and complete picture of each individual’s disease status.

Circulating tumor cells (CTCs) remain largely unexplored in the pediatric and adolescent/young adult (AYA) sarcoma population. Liquid biopsy is of particular interest for use in children as it avoids the need for repeated imaging and frequent sedation. Nonetheless, the scant few existing studies mostly focus on the detection of CTCs through the identification of specific fusion transcripts using rtPCR, a technique that is limited to patients in whom these transcripts are present (4, 5). Other studies focused on EPCAM expression, which varies depending on the type of sarcoma (6). Thus, to date, there is no truly universal marker for pediatric sarcoma.

CTCs can be difficult to characterize owing to their rarity; as few as 1 CTC can be found per 10 (7) blood cells (8). Many CTC capture technologies, including the FDA-approved Cellsearch are only capable of isolating CTCs that express epithelial cellular adhesion molecule (EPCAM) (7, 9). Sarcomas, however, do not generally express this protein and their CTCs cannot be isolated in this manner. Our previous research showed that cell-surface vimentin (CSV) is a universal marker of CTCs (10). Vimentin is expressed in healthy cells solely in the cytoplasm, with the exception of leukocytes which can express CSV (11). We, therefore, developed an automated microfluidics system to isolate and enumerate CSV^+^ CTCs from pediatric sarcoma patients’ peripheral blood.

Automation of CTC enumeration ensures reproducibility, allowing diagnostic laboratories to use our technology for these purposes. Here, we present the first study isolating CTCs using automated capture in pediatric and AYA patients with sarcoma. The primary aim of this study was to use CSV antibody capture to isolate CTCs from patients with various sarcomas. We further demonstrate that patients with active sarcomas have more CTCs than do long-term survivors. We additionally demonstrate the ability of our technology to distinguish between patients with active sarcomas and long-term survivors with a high degree of sensitivity and specificity. Finally, we demonstrate, using a combination of molecular data and CSV^+^ CTC enumeration, that the combined lack of CSV^+^ CTCs and lack of gene variants, amplifications, and fusions is associated with a clear survival advantage.

## Materials And Methods

### Patients and Data

This study was approved by the Institutional Review Board at The University of Texas at MD Anderson Cancer Center (Protocol: PA13-0014). All patients with a confirmed diagnosis of sarcoma at any disease stage who presented to our Pediatric and Adolescent and Young Adult service between January 2014 and January 2020 were eligible for enrollment. Patients with a confirmed sarcoma who had completed treatment at least 5 years before enrollment and had no evidence of disease were used as controls. Patients who had received chemotherapy within 30 days of sample collection were excluded from the analysis. Written informed consent was obtained from all the participants in this study. Peripheral whole blood samples were collected from 72 patients with active disease and 20 controls. CTCs were isolated from these samples as previously described.

### Manual Isolation of CTCs

Blood samples were subjected to gradient centrifugation by Ficoll-Paque (GE, Uppsala, Sweden). The buffy coat was then resuspended in 1 mL of RBC lysis buffer (Alfa Aesar, Haverhill, MA) for 7 minutes at room temperature. The resulting mixture was then resuspended in 2% FBS/PBS. The buffy coat was then subjected to the EasySep human CD45 Depletion Kit II (EasySep, Vancouver, Canada) for negative selection of CD45^+^ cells. Next, the cells were subjected to the EasySep Magnetic Isolation Kit for positive selection of CSV^+^ cells, using 84-1 antibody (MD Anderson, Houston, TX) to CSV.

### Automatic Isolation of CTCs

Blood samples were subjected to gradient centrifugation by Ficoll-Paque. The buffy coat was then resuspended in 100 μL of PBS containing 2% FBS. Four microliters of leukocyte aggregation inhibitor (Abnova, Taipei, Taiwan) was added to the resuspended cells. Next, Abnova Cytoquest slides were coated with 1 mg/mL of streptavidin for 1 h. The slides were then coated with an antibody to CSV (Abnova) for 1 h at room temperature, then washed 3 times with 200 μL of PBS. The cell suspension was then loaded into an Abnova Cytoquest microfluidics pump and pumped through the anti-CSV antibody-coated Abnova Cytoquest slide.

### Staining of CTCs

CTCs were blocked in 1% BSA containing an FcR blocking reagent (Abnova, Taiwan) for 30 minutes at room temperature. Cells were then resuspended in 1% BSA containing FcR blocker with 25 µg/mL CSV conjugated to FITC (Abnova, Taiwan) and 50 µg/mL anti-CD45 conjugated to PE for 1 h (Abnova, Taiwan). Cells were then counterstained with Hoechst prior to imaging.

**Figure 1:**
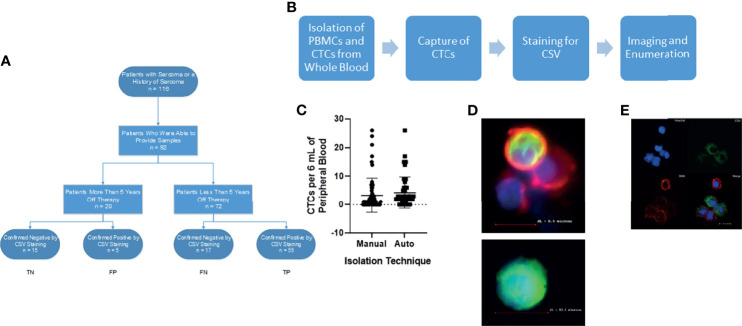
Isolation of CSV^+^CTCs from Sarcoma Patients. **(A)** STARD reporting diagram of consented patients. **(B)** Workflow diagram **(C)** Two-tailed t-test comparing automated *vs* manual technique for isolation of CTCs. *p* = 0.42 **(D)** Upper: Representative image of PBMC found during capture. Lower: Representative image of CSV^+^ CTC for capture. Cells were stained for nucleus (Blue), CSV (Green) and CD45 (Red) **(E)** Representative image of CSV^+^ CTC for validation. Cells were stained for nucleus (Blue), CSV (Green) and smooth muscle actin (Red).

Smooth muscle actin replaced CD45 staining in one instance of staining, shown in **Figure 1E**. Following automatic isolation, cells were blocked in 1% BSA containing an FcR blocking reagent for 30 minutes at room temperature. Cells were then resuspended in 1% BSA containing FcR blocker with 25 µg/mL anti-CSV conjugated to FITC.). Cells were then permeabilized with 0.25% NP40 (Amersham, Cleveland, OH) for 1 hour at room temperature. Cells were stained with 20 µg/mL anti-Smooth Muscle Actin conjugated to PE for 1 h (Abcam, UK) and then stained with 4 µg/mL goat-anti rabbit IgG (Invitrogen, Waltham MA) for 1 hour. Cells were then counterstained with Hoechst prior to imaging.

### Imaging

Slides were imaged on either a Keyence or a Bioview automated fluorescence microscope. Enumeration of CSV^+^ cells was performed manually on the Keyence microscope. CSV^+^CD45^-^ cells larger than 10 μM were deemed CTCs.

### Statistical Analysis

All statistical analyses were conducted using software GraphPad Prism 6 (San Diego, California) and R (R Development Core Team, Version 3.6.3). Continuous variables were summarized using means and standard deviations. Categorical variables were summarized using frequencies and percentages. Chi-squared tests were used to determine differences in CSV^+^CTC positivity based on age, gender, and tumor types. Non-parametric two-tailed unpaired t-tests were used to compare differences in CSV^+^CTCs per 6 mL of blood between groups. Wilcoxon rank-sum test was used to compare CTC counts between manual *versus* automated CTC capture methods, as well as between active sarcoma patients *versus* long-term survivors. The sensitivity and specificity of CTC counts for classifying patients with active sarcoma *versus* long-term survivors were assessed using receiver operating characteristic (ROC) analysis. The area under the ROC curve (AUC) was assessed and reported. The optimal cut-off points for CTC counts were obtained using the Youden Index method. Kaplan-Meier survival analyses and log-rank tests were used to compare overall survival between different groups according to presence of CTC and sarcoma-associated genetic mutations. In particular, the CTC counts were categorized as 0 (negative) *vs* >0 (positive). We also considered different groups based on the combination of CTC positivity and presence of genetic mutation, including CTC+mutation+, CTC+mutation-, CTC-mutation+ and CTC-mutation-. All of the statistical tests were two-sided. A p-value less than 0.05 were considered statistically significant.

## Results

### Patient Characteristics

This study enrolled 72 patients with sarcoma and 20 long-term survivors. The STARD reporting diagram is given in **Figure 1A**. The patients’ clinical characteristics and sarcoma types are summarized in **Table 1**. The median age of the patients was 14 years; 18 (20%) patients were 10 years old or younger. We found no differences in CTC detection based on age, sex, or type of tumor (**Table 1**).

**Table 1:** Patient characteristics.

Characteristic	
Age (median age = 14)	*p* = 0.3436
Age ≤ 10	18 (24%)
Age > 10	74 (76%)
Gender	*p* = 0.5212
Male	49 (53%)
Female	43 (47%)
Tumor Type	*p* = 0.7551
Osteosarcoma	26 (29%)
Ewing’s Sarcoma	10 (11%)
Rhabdomyosarcoma	16 (17%)
Nonrhabdomyosarcoma Soft Tissue Sarcoma	40 (43%)

### Manual and Automated Capture of CSV^+^CTCs Yields Similar Results

Our previous research demonstrated the utility of a CSV-targeted antibody in the isolation and identification of CTCs, in that CSV is a universal marker of CTCs (10, 12–15). We initially used the manual isolation method described previously to capture and image CTCs (10). To ensure that our technology could be used in CLIA-certified labs, we developed an automated technique (**Figure 1B**) to ensure reproducibility of results. In a similar manner to the manual CSV^+^ CTC process, the automated method can capture and isolate CTCs in the blood of patients with any type of sarcoma (**Figure 1D**). To ensure the consistency of these 2 sets of data, we performed statistical comparisons. As shown in **Figure 1C**, the two processes did not significantly differ in their ability to isolate and enumerate CTCs.

### CSV^+^ Cells Are CTCs

We previously showed that the captured CSV^+^ CTCs are tumor cells *via* FISH analysis, tumor cell spike assays, and some sequencing analyses (10, 14, 16). To further validate these observations for this study, we previously stained the CTCs of an angiosarcoma patient with CD31, an angiosarcoma biomarker, and reinforced this argument by staining the CTCs of a patient with embryonal rhabdomyosarcoma with smooth muscle actin (**Figure 1E**) (17). Expression of CD31 and smooth muscle actin, respectively, confirmed that the CSV^+^ cells observed were indeed angiosarcoma and embryonal rhabdomyosarcoma CTCs. These data, together with the oncogene amplification we reported in our previous publications, confirmed that the CSV^+^ cells captured by our technology were indeed tumor cells (10, 14).

### CSV^+^ CTCs Are More Abundant in the Blood of Patients With Active Sarcomas Compared to Long-Term Survivors

**Figure 2:**
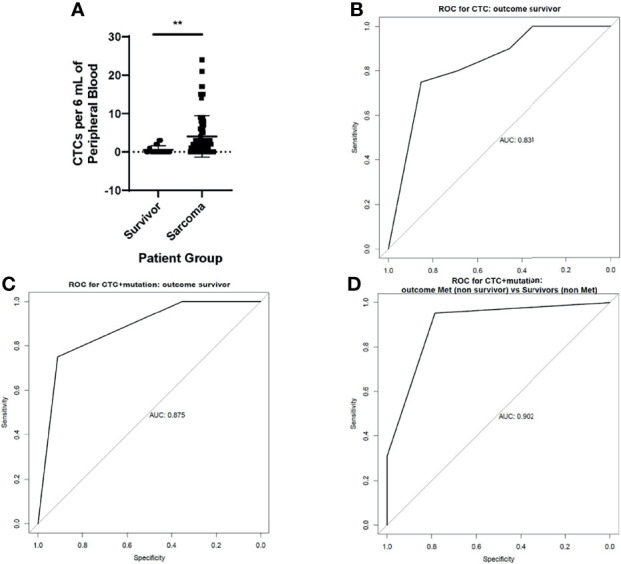
CSV^+^CTCs are elevated in the blood of patients with active sarcomas **(A)** Two-tailed t-test comparing CSV^+^CTCs per 6 mL of blood in long-term survivors *vs* patients with active sarcoma. *p* < 0.0001 **(B)** ROC curve generated based on CSV^+^CTCs per 6 mL of blood in long-term survivors *vs* patients with active sarcomas **(C)** ROC curve generated based on CSV^+^CTCs per 6 mL of blood in long-term survivors *vs* patients with active sarcomas, accounting for genetic variants **(D)** ROC curve generated based on CSV^+^CTCs per 6 mL of blood in long-term survivors *vs* patients with active sarcomas, accounting for genetic variants and metastasis.

With this information, we were able to enumerate CSV^+^ CTCs in the blood of pediatric and AYA patients with sarcoma. Sarcoma patients who were in remission for at least 5 years (long-term survivors) formed a control group. We found that patients with active sarcoma had significantly more (*p* < 0.0001) CSV^+^ CTCs per 6 mL of blood than did long-term survivors (**Figure 2A**). A range of 0 to 26 CSV^+^ CTCs per 6 mL of blood were found in these patients. Of the 72 patients with active sarcoma, 17 had no detectable CSV^+^ CTCs. Of the 20 control samples, 5 contained CSV^+^ CTCs, though the number of CTCs was low, ranging from 0 to 3 CTCs per 6 mL of blood.

### Sensitivity and Specificity of CSV^+^ CTCs for Detecting Sarcoma

We next constructed a receiver operating characteristic (ROC) curve using this information (**Figure 2B**). The area under the curve (AUC) of the ROC curve was 0.831. Because the Youden *J* value was 1 or more CTCs per 6 mL of blood, this level was used as the cutoff for CTC positivity. Using this cutoff value, the sensitivity and specificity of the test were 75% and 85.3%, respectively.

To further improve the test’s sensitivity and specificity, we added the results of genetic biomarker testing obtained during standard care. The rationale for adopting this strategy was that our recent report found that including the genetic biomarker *MYCN* to CSV+ CTC positivity boosted the accuracy of predictions of non-relapse from 95% to 100% in neuroblastoma patients who were in remission and receiving maintenance therapy (12). Because it is difficult to pinpoint a single mutation as a marker of sarcoma, we used the entire panel of genetic analysis results obtained as part of the standard of care to detect its impact on the ROC of CSV^+^ CTCs. Indeed, when the ROC curve took also took the genetic mutations listed in **Table 2** into account, the AUC increased to 0.875 (**Figure 2C**), with maximum combined sensitivity and specificity of 75% and 91.2%, respectively.

**Table 2:** Mutations examined.

Genetic Mutation	Sarcoma
FGFR4 + TP53	Rhabdomyosarcoma
EWSR1-CREB3L1	Sarcoma
ESWR Rearrangement	Rhabdomyosarcoma, Ewing’s Sarcoma
CDK6	Rhabdomyosarcoma
NF1	Rhabdomyosarcoma
TP53	Osteosarcoma, Rhabdomyosarcoma, Wilm’s Tumor, Teratoma

Because metastatic sarcoma with genetic mutations indicates a high disease burden, we constructed the ROC curve to compare patients with metastatic sarcoma to those for long-term survivors using the combination of genetic mutations and CSV+CTC. The AUC increased to 0.902 (**Figure 2D**).

### CSV^+^ CTC Positivity Is Associated With Poorer Overall Survival

**Figure 3:**
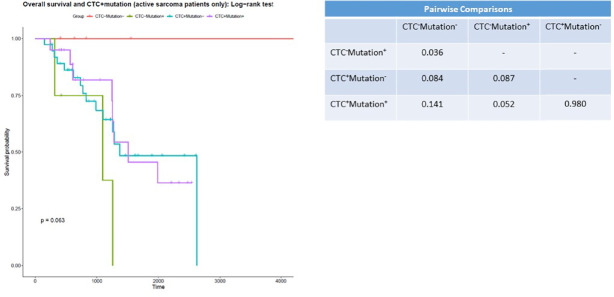
CSV^+^CTCs positivity is associated with worse prognosis Kaplan-Meier survival curve of patients with 0 CSV^+^CTCs with and without genetic variants *versus* patients with 1 or more CSV^+^CTCs with and without genetic variants. *p* = 0.063.

Again, we incorporated genetic mutation data (**Table 2**) into the survival analysis, as this could reveal subsets of patients who require less-aggressive therapies (**Figure 3**). We excluded long-term survivors from this analysis. When accounting for genetic mutations, we again found that patients with no genetic mutations and patients with no CSV^+^ CTCs and no sarcoma-associated genetic mutations had the longest survival times; all patients in this category survived. We additionally found that patients who were CSV^+^ CTC negative and mutation positive had the lowest median survival time, 1096 days. CSV^+^ CTC-positive patients without any sarcoma-associated genetic mutations had a median survival time of 1372 days, while CSV^+^ CTC-positive patients bearing sarcoma-associated gene variants had a median survival time of 1509 days. Of note, only a comparison between CTC negative patients with and without mutations yielded significance.

We additionally examined differences in CSV^+^CTCs per 6 mL of blood in patients in an attempt to determine whether we could use our test to distinguish between patients with non-metastatic *vs* metastatic sarcomas (**Supplemental Figure 1A**). However, we were unable to find any differences between the two groups in total CSV^+^CTCs per 6 mL of blood.

## Discussion

To date, this is the largest study to evaluate CTCs in pediatric and AYA patients with sarcoma. The wide variety of sarcomas included in this study offers additional confirmation of CSV as a marker of sarcoma CTCs, especially in pediatric patients (10). Our finding that CSV was expressed in a cluster with SMA further confirms our belief that CSV is a CTC-specific biomarker for cancer (18, 19). Our previously published data also showed that CSV^+^ CTCs bear amplified oncogenes *TP53, MDM2*, and *KRAS* (10). This is associated with metastasis and tumor heterogeneity in sarcoma and with a poor prognosis (20, 21). These findings confirm our belief that CSV^+^CTCs are indeed CTCs.

Tissue biopsy is considered the gold standard for diagnosis of sarcoma. However, it is an invasive procedure and carries risks such as contamination and needle tract seeding (22). We observed more CSV^+^ CTCs in patients with active sarcomas than in long-term survivors. This was expected, as long-term survivors likely have no tumors from which CTCs can shed. Although long-term survivors had fewer CSV^+^ CTCs than did patients with active sarcomas (**Figure 2A**), there were numerous long-term survivors who had blood in which we found CSV^+^ CTCs. The presence of these cells, although at a lower percentage, in survivors indicates that further research may be necessary before these cells can be used in diagnosis. A possible reason for the presence of CSV^+^ CTCs in the blood of survivors may be false positives. It has been shown that activated macrophages, apoptotic neutrophils and virally infected cells can both express CSV (17, 23, 24). All three of these cell types are larger than 10 uM in diameter, but only virally infected cells are CD45 negative. It is possible that these cells may contribute to the number of CSV^+^ CTCs we enumerated in survivors. A potential remedy for this would be to stain for sarcoma specific markers, such as smooth muscle actin alongside CSV and CD45.

Our analyses used a cohort of long-term sarcoma survivors as a control group. The ROC curve was able to distinguish between patients with active sarcoma and survivors. When genetic mutations were incorporated into the model, the AUC improved. However, recurrence is a perennial concern even after remission is achieved. Multiple publications have shown that early detection of CSV^+^ CTCs can predict relapse and recurrence in various cancer types including neuroblastoma and breast, prostate, and colorectal cancers (12, 25–27). Examining CSV^+^ CTCs in the blood of sarcoma patients in remission would allow a way of predicting relapse other than the existing standard of surveillance imaging.

Our automated method for isolating and enumerating CSV^+^ CTCs showed no significant differences in the total number of CSV^+^ CTCs detected per 6 mL of blood, nor were the variances between the 2 groups significant (*p* = 0.5578). We chose the cutoff of 1 or more CSV^+^ CTCs per 6 mL of blood to indicate positivity, as this was the value with the highest combined sensitivity and specificity. The automated procedure will also advance our goal of using CSV^+^ CTCs as a prognostic tool, as it is CLIA compliant.

CSV^+^ CTC positivity was indicative of poorer overall survival. We additionally found no deaths among the patients who had no genetic risk factors and no CSV^+^ CTCs. All other patient groups had median survival times under 1600 days. These findings suggest that a combination of molecular testing and CSV^+^ CTC enumeration can reveal a subset of patients that could safely undergo less-aggressive treatment. Furthermore, when we incorporated molecular testing data into our results, we found that patients who were both CSV^+^ CTC negative and tested negative for any variant genes had a survival advantage compared to all other groups. No members of this group died. Intriguingly, we found the greatest difference in survival in patients who were mutation negative. This suggests that this subset of patients may receive the most benefit from our assay. Unfortunately, the pairwise comparison between these groups did not reach significance (*p* = 0.084).

A comprehensive panel that includes both testing for the variant genes listed in **Table 2** and CSV^+^ immune cells could allow patients with negative results on both these tests to discontinue therapy earlier, limiting both the deleterious effects of chemotherapy and patient anxiety.

In summary, this study demonstrates the utility of using CSV in pediatric and AYA patients as a biomarker to detect CTCs. Our findings demonstrate the prognostic value of CSV^+^ CTCs, which may allow the early identification of patients who may benefit from modified therapies. While further research is needed, the enumeration of CSV^+^ CTCs in pediatric and AYA patients with sarcoma may have therapeutic and prognostic implications that may help guide patient management in the future.

## Data Availability Statement

The raw data supporting the conclusions of this article will be made available by the authors, without undue reservation.

## Ethics Statement

The studies involving human participants were reviewed and approved by the Institutional Review Board at The University of Texas at MD Anderson Cancer Center (Protocol: PA13-0014). The patients/participants provided their written informed consent to participate in this study.

## Author Contributions

LD and DR performed the experiments, were responsible for data curation and wrote the manuscript. IB and AS performed the experiments. JF, WZ, AA were responsible for data curation. JW and WH performed statistical analyses. JG, DL, NG, DH, CH, EK, RG, and ND developed the concept for the manuscript. SL developed the concept for manuscript, conceived the experiments and edited the manuscript. All authors approved the final manuscript as submitted and agree to be accountable for all aspects of the work.

## Funding

This study was funded by the NIH Grant R01 EB026291. The NIH had no role in the design and conduct of this study.

## Conflict of Interest

The authors declare that the research was conducted in the absence of any commercial or financial relationships that could be construed as a potential conflict of interest.

## Publisher’s Note

All claims expressed in this article are solely those of the authors and do not necessarily represent those of their affiliated organizations, or those of the publisher, the editors and the reviewers. Any product that may be evaluated in this article, or claim that may be made by its manufacturer, is not guaranteed or endorsed by the publisher.

## Supplementary Material

Supplementary Figure 1 There is no significant difference between CSV^+^CTCs per 6 mL of blood in nonmetastatic *vs* metastatic patients. **(A)** Two-tailed t-test comparing CSV^+^CTCs per 6 mL of blood in non-metastatic *vs* metastastic patients. *p* = 0.15.

## Abbreviations

CSV, Cell Surface Vimentin; AYA, Adult and Young Adolescent; ROC, Receiver Operating Characteristic Curve; AUC, Area Under Curve; CTC, Circulating Tumor Cell; EpCAM, Epithelial Cellular Adhesion Molecule; CLIA, Clinical Laboratory Improvement Amendments.
