# Supplementary figures and images for: Prognostic Value of Cell-Surface Vimentin-Positive CTCs in Pediatric Sarcomas
Source: Front Oncol. 2021 Dec 9;11:760267. doi: 10.3389/fonc.2021.760267 (PMC8695931; doi:10.3389/fonc.2021.760267)

# Supplemental Figure 1

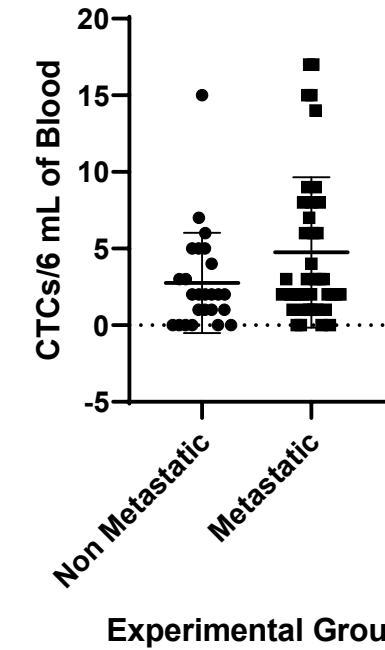

**Non Metastatic**

**Metastatic**

## Experimental Group

Supplement: Supplementary Figure 1 — There is no significant difference between CSV+CTCs per 6 mL of blood in nonmetastatic vs metastatic patients. (A) Two-tailed t-test comparing CSV+CTCs per 6 mL of blood in non-metastatic vs metastastic patients. p = 0.15. [file DataSheet_1.pdf]
